# Traditional antidiabetic medicinal plants of Himachal Pradesh: ethnomedicinal evidence and drug-discovery potential

**DOI:** 10.3389/fnut.2026.1804763

**Published:** 2026-05-25

**Authors:** Mehak Thakur, Szilvia Várallyay, Brigitta Tóth, Diána Ungai, Tejas Suthar, Béla Kovács, Endre Harsányi, Marek Zdaniewicz, Ayaz Mukarram Shaikh, Asgar Ali

**Affiliations:** 1 School of Biological and Environmental Sciences, Shoolini University of Biotechnology and Management Sciences, Solan, Himachal Pradesh, India; 2 Faculty of Agriculture, Food Science, and Environmental Management, Institute of Food Science, University of Debrecen, Debrecen, Hungary; 3 Research and Innovation Center, University of Nyíregyháza, Nyíregyháza, Hungary; 4 Independent Researcher, Chicago, IL, United States; 5 Agricultural Research Institutes and Academic Farming (AKIT), Faculty of Agriculture, Food Science, and Environmental Management, University of Debrecen, Debrecen, Hungary; 6 Department of Fermentation Technology and Microbiology, Faculty of Food Technology, University of Agriculture in Krakow, Krakow, Poland; 7 Department of Food Science and Technology, Graphic Era (Deemed to be University), Dehradun, Uttarakhand, India; 8 Department of Integrative Agriculture, College of Agriculture and Veterinary Medicine, United Arab Emirates University, Al-Ain, United Arab Emirates

**Keywords:** diabetes, ethnobotany, ethnopharmacology, medicinal plants, phytoconstituent

## Abstract

Diabetes mellitus is a rapidly increasing lifestyle disorder posing a serious global health threat. This review specifically focuses on indigenous medicinal plants of Himachal Pradesh, India, a region rich in ethnomedicinal diversity. The primary objective of this review is to systematically compile and analyze the ethnomedicinal, phytochemical, and pharmacological evidence of antidiabetic plants used in this region. Data were collected from various scientific sources including Scopus, PubMed, ScienceDirect, Google Scholar, ResearchGate, books, and other relevant literature from published studies spanning the period from 1995 to 2025. According to literature, 80 ethnomedicinal herbs are believed to be effective in treating diabetes. For the preparation of natural remedies, different plant parts were often used such as roots (17%), leaves (24%), fruits (15%), bark (13%), whole plants (3%), and flowers (3%). These plants are widely used in the traditional healthcare practices of Himachal Pradesh. Furthermore, the review highlights traditionally used medicinal plants for diabetes management, along with the reported phytochemicals responsible for their antidiabetic activity. Moreover, available preclinical *in-vivo* studies on selected plants have been included to support their therapeutic potential. Furthermore, we suggest that future studies should include molecular docking analysis of the reported antidiabetic phytochemicals to better understand their mechanism of action at the molecular level. Additionally, conservation and sustainable utilization of these indigenous plant resources are crucial for future therapeutic applications.

## 1 Introduction

Nearly 80% of the population is projected to rely on plant-based remedies to treat various disorders as they are safe, cost-efficient, easily accessible, and extremely effective (1). India is also known as the “botanical garden of the world” due to its tremendous diversity, and much more than 70% of its rural areas rely on traditional plant medicines (2). Himachal Pradesh (HP) is a hilly state, with latitudes ranging from 30°22′44′′ N to 33°12′44″N and longitudes ranging from 75°45′44″E to 79°04′20″E (3). HP is endowed with an abundance of plant diversity, as well as medicinal properties and other valuable species (4).

The medicinal flora has developed an extensive variety of therapies, including diabetes mellitus (DM), a disease whose “killer” nature and epidemic severity pose a challenge to humanity (5). The disease rate is expected to reach 366 million by 2030, primarily in China and India. Among diabetics, there are three types: type 1 diabetes (T1DM), type 2 diabetes (T2DM), and gestational diabetes (GDM) (6). Diabetic complications are most prevalent among people with type 2 diabetes, which is characterized by hyperglycemia oxidative stress, and hyperlipidemia, which can cause chronic problems with the nerves, eyes, blood vessels, and kidneys (7).

Several traditional medicines and plants are well-known for delaying and preventing the development of diabetes-related problems and changing metabolic imbalances (8). Furthermore, the existence of phytoconstituents, plants have a larger potential than manufactured medications (9). One of the primary benefits of using plants is that they do not have negative side effects that are frequently associated with other medications (1). All the drugs have a significant mechanism, limited efficacy, and tolerability (10). Over the last decades, there has been an immense interest in discovering the secrets of natural treatments due to biological screening, isolation, and clinical trials of plants that have been developed for this purpose (11). Around 1,200 herbal treatments for diabetes have been reported; 343 species were studied for their capability to lower sugar level, and 656 species of flowering plants are usually used for the treatment of diabetes in Asian cultures (8).

The Ayurvedic Pharmacopeia Committee includes 350 plant species (Government of India), of which 225 species are found in Himachal Pradesh and are commercially available (12). Various medicinal plants are extensively described in the Rigveda (2500–1800 BC), Charaka Samhita, and Sushruta (13, 14). Potential phytochemicals that possess drug-like and pharmacophore properties could provide a novel scaffold for combinatorial preparations (15). As there are certainly available medications, diabetes patients still struggle to maintain adequate hyperglycemia control due to continuing declines in *β*-cell function (16).

Nowadays ethnomedicinal information that is orally passed down through generations is at risk so it can be forgotten or lost forever (17). However, this information can be retained, preserved, and sustainably applied for diagnosing and inhibiting diabetes in humans in the long run as mentioned in Table 1. New drugs or medicines can be created using this knowledge of medicinal plants. This review article aims to focus on the mechanisms of phytochemicals used as prototypes for synthesizing new molecules to lower glucose level and enhance insulin secretion. In contrast to a simple review article, this work delves into mechanisms that could provide an insight into how medicinal plants could be used for the treatment of diabetes based on scientific literature. It is known that natural products derived from medicinal plants like flavonoids, alkaloids, and terpenoids act in an antidiabetic manner by increasing insulin secretion, increasing insulin sensitivity, and reducing glucose uptake in the intestines. Moreover, medicinal plant-derived phytochemicals possess antidiabetic potential through multi-target mechanisms and can be further validated using molecular docking to understand their interaction with key proteins. This review incorporates ethno-medicinal information, phytochemistry, and *in-vivo* studies of medicinal plants from Himachal Pradesh for antidiabetic therapy, providing valuable scientific insight.

**Table 1:** A list of most common medicinal herbs used for the management of diabetes.

Botanical name	Common name	Plant part used	Mode of application	Place	References
*Acacia arabica* (Lam.) Willd.	Kikar, Babul	Gum	As a decoction	Hamirpur	(48)
*Acacia nilotica* (L.) Delile	Kikar	Flower, leaves and seeds	Used decoction, powder, orally and Juice forms	Mandi, Sirmaur, Solan and Una	(9)
*Aconitum heterophyllum* Wall. ex Royleall. ex-Royle	Atish, Patish	Roots	Aa a decoction	Shimla	(49)
	Atish and Patish	Whole plant	Root extract is taken orally three times in each day	Lahaul and Spiti	(50)
*Acacia nilotica* (L.) Delile	Bil and Blpatri	Leaves	Juice, decoction, and powder	Hamirpur	(48)
*Aegle marmelos* (L.) Corrêa	Dhardu	Fruit and leaves	Decoction and as a juice	Hamirpur	(51)
*Ajuga bracteosa* Wall. ex Benth	Neelkanthi/Jwara	Leaves	About 5–10 leaves are taken	Chamba	(52)
*Bulga parviflora* (Benth.) Kuntze	Neelkanthi	Leaves	Juice	Una	(53)
	Neelkanthi	Leaves	Fresh leaves are chewed	Shimla	(54)
*Aconitum heterophyllum* Wall. ex-Royle	Kalasiri	Bark, flowers, and roots	Decoction	Bilaspur, Chamb, Hamripur, Sirmaur, Solan and Una	(9)
*Allium sativum* L.	Lahusan	Bulb	Bulb of the garlic is used as diabetes	Hamirpur	(48)
	Lahasun	Bulb	Bulbs were used to treat diabetes	Solan	(50)
*Aloe barbadensis* Mill.	Kabbarya	Leaves	Juice, decoction, and powder	Hamirpur	(48)
*Anogeissus latifolia* (Roxb. ex-DC.) Wall. ex Bedd.	Dhawa, Dhaura	Bark, flowers, fruits, roots, and stems	Powder form	Hamripur, Sirmaur, Solan and Una	(9)
*Argemone mexicaa* L.	Bharbhand	Root	Root powder is used	Hamirpur	(48)
*Asparagus adscendens* Roxb.	Sanspai	Roots	Decoction and as a juice	Hamirpur	(51)
*Asparagus racemosus* Will	Sahanspai	Roots	Taken as powder	Una	(5)
	Shatavri	Rhizome and aerial part	Oral	Kangra	(55)
*Azadirachta indica* A. Juss.	Neem	Leaves	Juice, decoction, and powder	Hamirpur	(48)
*Begonia picta* Sm	Pethu	Seeds and fruit	Directly eaten	Mandi	(51)
	Pethu	Pulp, seed, and fruits	Decoction and as a juice	Hamirpur	(51)
*Berberis asiatica* Roxb. ex-DC.	Kashmal	Roots	Decoction	Mandi	(50)
*Berberis lycium* Royle	Kasmal	Roots	Decoction	Una	(53)
*Bidens pilosa* L.	Gumber	Leaves	Decoction and powder of leaves is used diabetes	Solan	(56)
*Bougainvillea glabra* Choisy	Bouganbael	Leaves	Decoction of leaves	Kangra	(55)
*Bougainvillea spectabilis* Willd.	Booganbel	Whole plant	Directly eaten	Solan	(56)
*Calotropis gigantea* (L.) Dryand.	Aak	Bark, roots	Powder of bark with hot water	Kangra	(55)
*Cassia fistula* L.	Karen and Amaltas	Bark	Powder is used	Hamirpur	(48)
*Cassia occidentalis* L.	Baru, elwan, relu Jamun	Seed	Seed powder and juice	Hamirpur	(48)
	Sadabahar	Roots and leaves	Decoction of roots and leaves	Solan	(56)
*Catharanthus roseus* (L.) G. Don	Patindu	Leaves	Fresh leaves are chewed	Una	(5)
*Cissampelos pareira* L.	Tarbuj	Fruits and seeds	Decoction	Kangra, Sirmaur, Solan, and Una	(57)
*Citrullus lanatus* (Thunb.) Matsum. & Nakai	Kunduri	Flowers, fruits, leaves and roots	Decoction	Una	(58)
*Coccinia grandis* (L.) Voigt	Jamun		Leaves taken orally	Chamba	(59)
*Corylus jacquemontii* Decne	Panja	Tubers	Decoction of fresh or dried tubers	Chamba	(60)
*Dactylorhiza hatagirea* (D. Don) Soó	Sisham	Leaves	Juice, decoction, and powder	Hamirpur	(48)
*Dalbergia abbreviata* Craib	Phunta	Leaves	Orally	Solan	(56)
*Erigeron annuus* (L.) Pers.	Koda	Seeds	As a decoction or powder	Shimla	(61)
*Eleusine coracana* (L.) Gaertn.	Chem, Macho, Pret, and Yamkand	Leaves	Young shoots are cooked and used as digestive.	Kullu, Sirmaur and Kinnaur	(58)
*Henningia himalaica* (Baker) A.P. Khokhr.	Ogla	Seeds and leaves	Both are boiled and eaten	Kinnaur, Shimla, Sirmaur, Solan and Una	(62)
*Ficus bengalensis* L.	Bar, Bargad, and Bor	Fruits, latex and leaves	Juice	Hamirpur, Shimla, Sirmaur, Solan, Una	(58)
	Bargad/Bar/Bat Vriksh	Young buds	Milky juice	Chamba and Hamirpur	(63)
	Bargad	Bark, Roots and leave	As a decoction or powder	Bilaspur	(64)
*Ficus glomerata* Roxb.	Gular and Umar.	Bark, leaves and latex	Juice	Kangra Sirmaur, Solan, Una	(58)
	Gular	Roots	Powder of dry roots	Chamba and Hamirpur	(63)
*Ficus racemosa* L.	Tarayamblu	Fruit, roots	Directly eaten	Mandi	(51)
		Fruits, roots, bark, and latex	Decoction and as a juice	Hamirpur	(51)
*Ficus religiosa* L.	Pipal	Bark, roots	Decoction of the roots and bark	Solan	(56)
*Gentiana kurroo* Royle	Karvi	Roots	Powder of roots	Shimla	(61)
*Gymnema acuminatum* Wall	Kadu	Roots	Root powder taken orally	Shimla	(61)
*Helicteres isora* L.	Gurmar	Roots	Powder of dry roots	Chamba and Hamirpur	(63)
*Ficus religiosa* L.	Marorphali	Bark	Decoction	Chamba and Hamirpur	(63)
	Marorphali	Bark, fruits, roots, seeds, and leaves	Decoction	Kangra Sirmaur Solan	(58)
*Hordeum vulgare* L.	Jau	Seed	Directly eaten	Mandi	(51)
		Seeds	Decoction and as a juice	Hamirpur	(51)
*Jasminum officinale* L.	Chameli	Leaves	Boiled leaves	Hamirpur and Kangra,	(65)
*Juglans regia* L.	Walnut	Fruit	Directly	Sirmaur	(50)
*Juniperus communis* L.	Aaraar	Fruit	Directly eaten	Lahaul and Spiti	(66)
*Mallotus philippensis* (Lam)	Kamala or Kabila	Fruit	Unripe fruits are found to be useful in diabetes	Chamba and Hamirpur	(63)
*Malus pumila* Mill.	Seb	Leaves	Decoction	Kullu	(56)
*Medicago polymorpha* L.	Maina	Leaves	Decoction	Kullu	(63)
*Momordica charantia* L.	Khukhni	Seeds	Sprouts of seeds used in diabetes	Hamirpur	(67)
*Momordica charantia* L.	Karela	Fruit	Cooked as vegetable	Hamirpur	(48)
	Karela	Fruit and seeds	Taken as vegetable	Hamirpur	(53)
*Momordica dioica* Roxb. ex Willd	Kakroon	Fruit	Cooked as vegetable	Hamirpur	(48)
*Morina alba* Hand.-Mazz.	Sainjna	Bark, leaves, roots, and seeds	Decoction	Kangra Sirmaur Solan and Una	(58)
*Moringa oleifera* Lam	Moringa	Leaves	Decoction	Mandi	(53)
*Morus alba* L.	Toot	Fruits	Directly eaten	Solan, Kangra, Chamba and Bilaspur	(68)
*Morus nigra* L.	Tut	Roots and leaves	Decoction of roots and leaves	Solan	(56)
*Murraya koenigii* (L.) Spreng	Kari patta	Leaves	Decoction and powder of leaves	Solan	(56)
*Musa paradisiaca* L.	Kela	Fruit	Unripe fruits are found to be useful in diabetes	Chamba and Hamirpur	(63)
*Nerium indicum* Mill.	Kaner	Bark, leaves, roots, whole plant	Directly eaten	Bilaspur, Sirmaur Solan and Una	(9)
*Opuntia stricta* (Haw.) Haw.	Nagphany	Roots	Powder of dry roots	Chamba and Hamirpur	(63)
*Paspalum scrobiculatum* L.	Kodo and Kodra	Roots, seeds, and stems	Decoction	Hamripur, Kangra and Una	(9)
*Picrorhiza kurooa*	Kavi	Roots	Root powder taken orally	Shimla	(69)
*Picrorhiza kurroa* Royle ex Benth.	Bhekal	Bark	Powdered of bark taken orally twice a day with lukewarm	Chamba	(59)
*Prinsepia utilis* Royle	Matter	Seeds	Taken as vegetable	Hamirpur	(53)
*Pogostemon amaranthoide* Benth	Kalibasuti	Leaves	Fresh leaves are chewed	Una	(5)
*Pongamia pinnata* (L.) Pierre	Karanja	Bark	Powder is used	Hamirpur	(48)
*Prunus armeniaca* L.	Karanjoatra	Leaves and fruits	As a decoction or powder	Bilaspur	(64)
*Punica granatum* L.	Anar	Fruit	In the form of powder	Hamirpur	(70)
*Rhododendron arboreum* Sm.	Burans	Leaves	Taken as powder		(70)
*Roylea cinerea* (D. Don) Baill.	Karway and Karu	Leaves	Chewing of leaves in the early morning	Mandi, Sirmaur, Shimla, Solan	(71)
*Sapindus mukorossi* Gaertn.	Ritha	Seeds	Seeds are eaten	Una	(5)
*Senegalia tenuifolia* (L.) Britton & Rose	Khair	Bark	Infusion of bark with hot water cure diabetes	Kangra	(55)
*Swertia chirayita* (Roxb.) Buch.-Ham. ex C. B. Clarke	Bhunimba, Chirata and Chirayta	Leaves	Taken as juice	—	(50)
*Syzygium cumini* (L.)	Jam	Bark, fruits, leaves and seeds	Orally	Chamba, Hamripur, Mandi, Sirmaur, Una	(9)
	Jamun	Seeds	Seeds powder and juice	Hamirpur	(48)
	Jamun	Leaves	Decoction and powder of leaves is used diabetes	Solan	(56)
	Jamun	Leaves and seeds	Directly eaten	Mandi	(51)
	Jamun	Bark, leaves and seeds	Decoction and as a juice	Hamirpur	(51)
	Jamun	Fruit and leaves	Leaves powder with tea is taken once a day in the morning for a few days	Bilaspur	(64)
	Jamun	Seeds	Decoction of seeds are used	Chamba and Hamirpur	(63)
*Terminalia arjuna* (Roxb. ex-DC.) Wight & Arn	Arjun	Leaves	Juice, decoction, and powder	Hamirpur	(48)
*Tinospora andamaa* Diels	Guduchi	Leaves	Decoction	Mandi	(53)
	Giloe, Gulje	Stem	Dried stem pieces are used	Hamirpur	(48)
	Giloe/Gulanj	Stem	Stem of juice is used	Chamba and Hamirpur	(63)
	Giloya	Stem	Decoction of stem	Kangra	(55)
	Giloye	Leaves	Decoction	Kullu	(56)
*Trigonellafoenum graecum* L.	Methi	Seed	Powdered seed is taken with cold water	Hamirpur	(48)
*Vinca rose* (L.) G. Don	Sadabahar	Leaves	Juice, decoction, and powder	Hamirpur	(48)
*Withania somnifera* (L.) Dunal.	Ashvagandha	Roots	Root powder is used	Hamirpur	(48)

## 2 Materials and methods

An extensive compilation of information on the use of ethnomedicinal plants in the treatment of diabetes disorder was compiled from a variety of sources, including literature, books, and relevant publications on Google Scholar, Research Gate, Science Direct, Scopus, and PubMed, etc. Detailed information on the collected plants is given in tabular form, including their botanical name. In this review, data were systematically collected from published studies spanning the period from 1995 to 2025. The plant species was identified and its nomenclature verified using the database available at https://www.plantlist.com/.

## 3 The current state and treatment of diabetes

**Figure 1:**
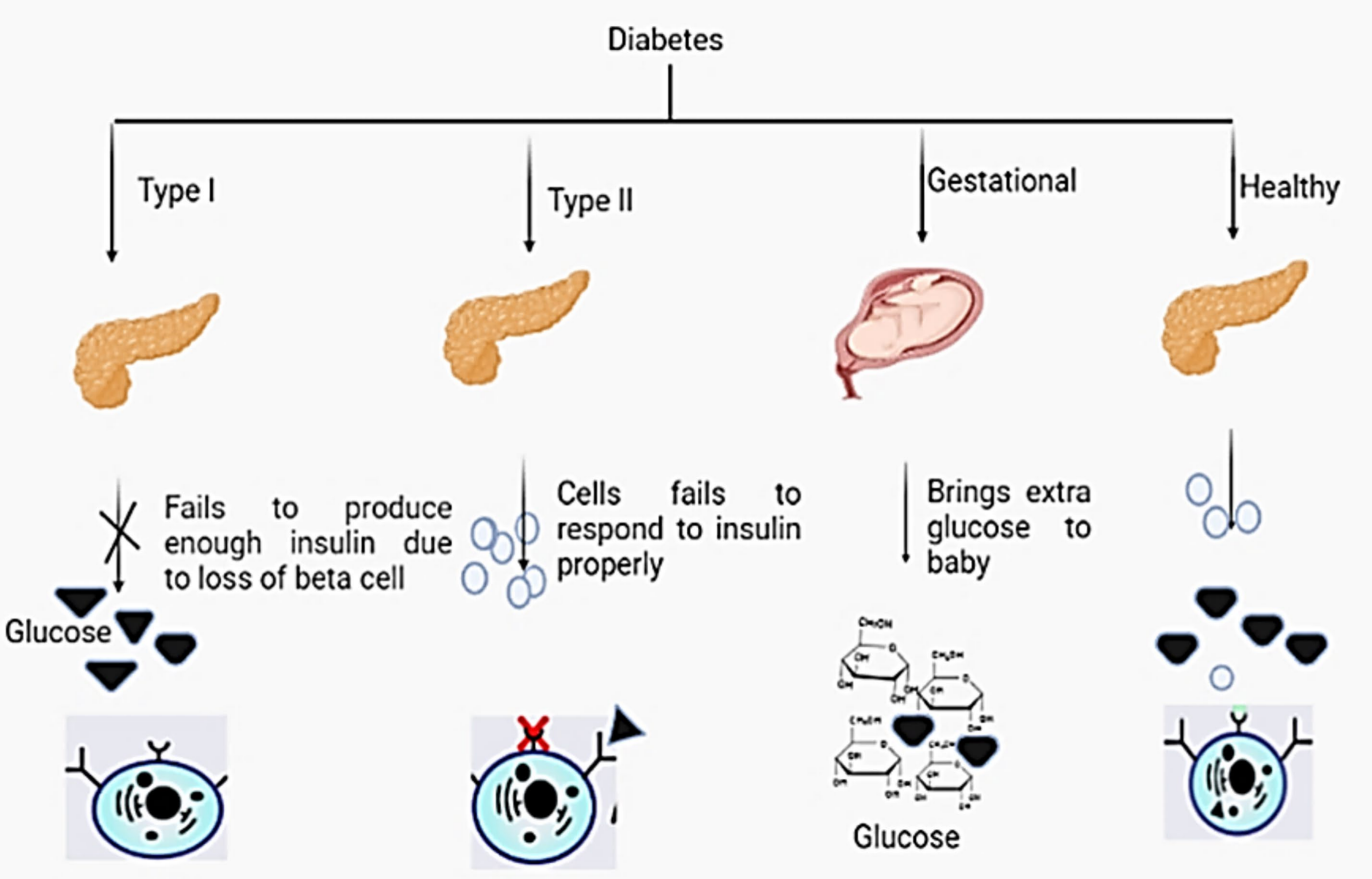
Categories of diabetes based on its pathophysiology.

Diabetes is characterized by hyperglycemia (high blood sugar) because of an inability to produce insulin. The inability of cells to produce insulin is known as insulin resistance (IR) (18). In addition to diabetes, IR has been linked to the development of metabolic syndrome and cardiovascular disease (19). T1DM is caused by the death of cells, resulting in a deficiency of insulin synthesis (20). T2DM is caused by insulin resistance and an inability to respond appropriately to hyperglycemia, and it affects most diabetics (90–95%) gestational diabetes is due to high level of sugar in the blood during pregnancy as described in Figure 1.

**Figure 2:**
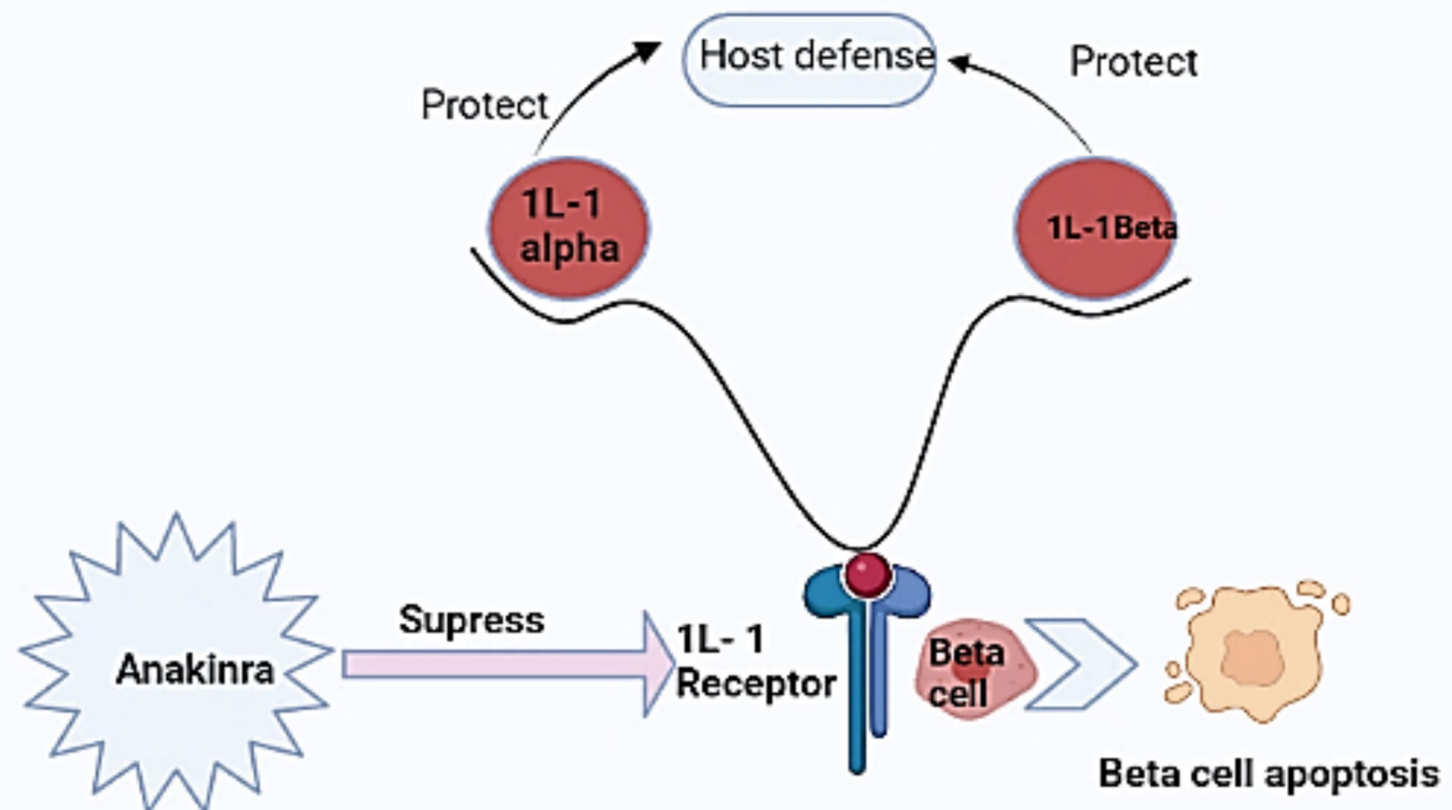
Treatment with interleukin-1–receptor.

According to an epidemiological study, DM is preceded by endocrine diseases (21). It is expected that diabetes will be one of the leading causes of death in the 21st century (22). Herbal components are the most prolific source of novel pharmacological entities, with diverse medicinal indications and chemical structures (23). Several diabetes treatments with disease-causing actions have already been identified (24). An example is treatment with anakinra that blocks the effects of interleukin-1α and β cells (25). Both forms of interleukin-I are produced by many cells (e.g., beta cells), play positive roles in host defense, and induce β -cell apoptosis (26). Treatment with an interleukin-I receptor antagonist is shown in Figure 2 and was permitted by the Food and Drug Administration in 2001 (27, 28).

The vast pervasiveness, progressive nature, variable pathogenesis, and repercussions of diabetes highlight the urgent requirement for effective therapies (29). Despite considerable advances in diabetes therapy, patient health outcomes are still far from satisfactory (30). Many approaches are recommended now as plants contain chemical compounds with anti-diabetic properties including glycoside, terpenoids, carotenoids, alkaloids, and flavonoids (31). The anti-hyperglycemic effects of plants are often attributed to their ability to restore pancreatic tissue function, achieved by enhancing insulin secretion and reducing glucose absorption in the intestine (32). Several forms of pharmacological investigation on anti-diabetic plants increased the number of individuals who used these natural chemicals to cure their conditions (28). Herbal remedies were used to treat diabetes before insulin and other blood sugar-lowering agents were discovered (14). A few of the most used plants have been covered in Table 1.

## 4 Role of plant parts in antidiabetic ethnomedicinal practices

**Figure 3:**
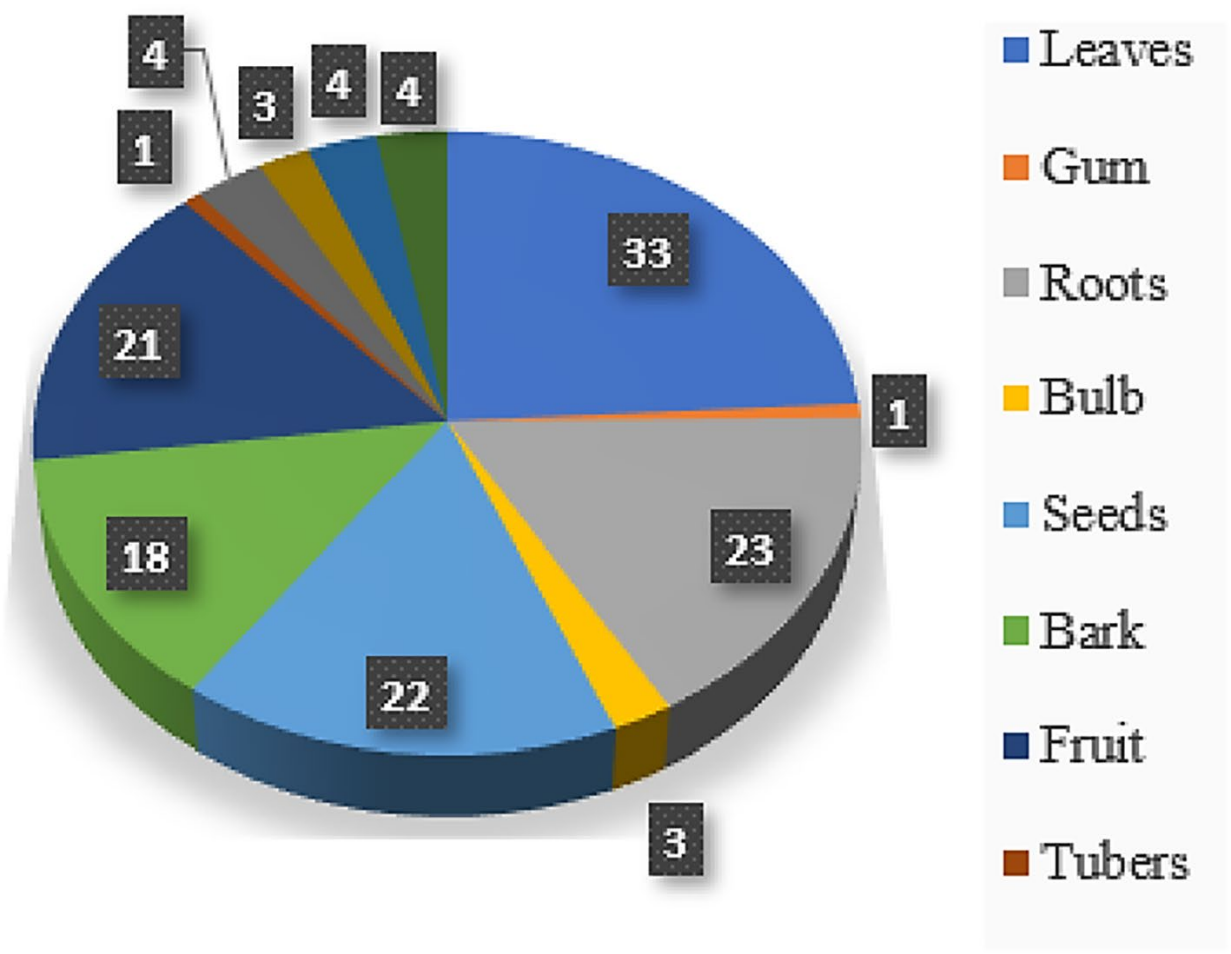
Analysis of the data showing a total assessment of plant components used for diabetes. Here number shows the number of plant part used for the treatment of diabetes. Here number present the quantity of plant part used for diabetes treatment.

Diabetes has been cured with phytoconstituents present in plant parts like bark, roots, flowers, fruits, leaves, seeds, and sometimes entire plants. This also increases local people’s utilization of these plants. All ethnomedicinal plants include phytochemical elements that may be beneficial for influencing illness. The traditional uses of plants have been demonstrated by a revaluation of their efficiency in different places. There are variations in the convention of plant components for lowering glucose level, which are illustrated in Figure 3.

## 5 Phytoconstituents present in the ethnomedicinal plants

**Figure 4:**
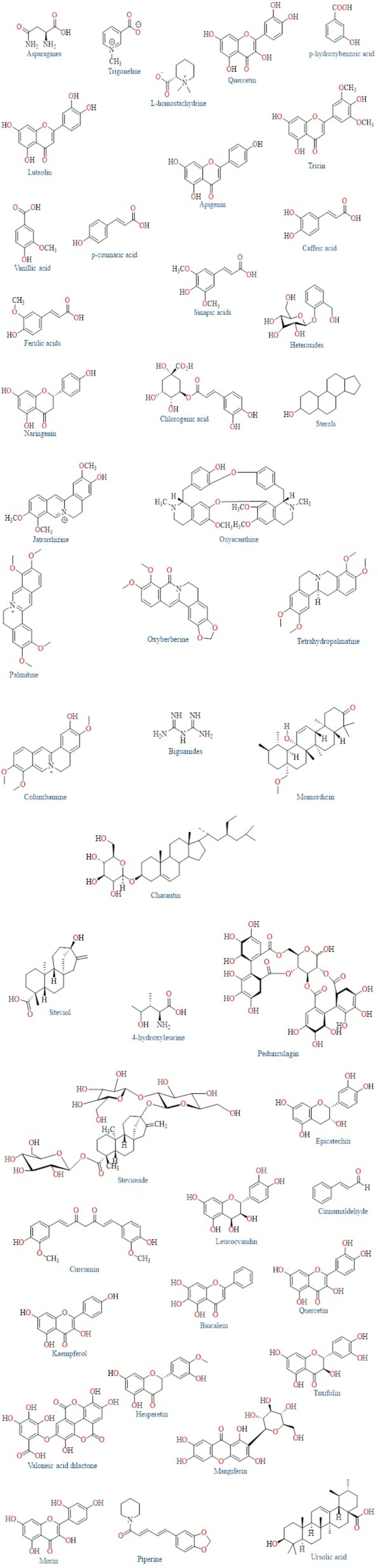
Chemical structures of major bioactive phytoconstituents which have antidiabetic activity. This structural diversity highlights the potential of plant-derived molecules as promising candidates for the development of novel antidiabetic therapeutics.

Many of the chemical compounds found naturally in plants that are used to treat diabetes are formed or stimulated by plants (33, 34). Therefore, this review describes numerous phytoconstituents of ethnomedicine plants used by the Himachal Pradesh population to treat diabetes (35). The plants are also used for the development of antioxidant and antimicrobial drugs (36). Researchers have become increasingly interested in medicinal plants over the past few years since secondary metabolites have been identified and their potential to prevent chronic and degenerative diseases has been proven (37). For example, phenolics are well-known secondary metabolites with numerous biological activities, like antioxidant, antidiabetic, antimutagenic, and anti-inflammatory (38). A significant amount of research is being conducted to discover strong anti-diabetic herbal medications using ethnopharmacological methods such as isolation and identification of active metabolites and their mechanisms of action (39). The chemical structures of major phytoconstituents, as reported in the literature to possess antidiabetic properties, are shown in Figure 4. Various other chemicals present in medicinal plants are mentioned in Table 2.

**Table 2:** Main phytochemicals present in ethnomedicinal plants with hypoglycemic or anti-diabetic activity.

Plant name	Phytochemical constituents	References
*Medicago sativa* L.	Aasparagines, trigoneline, stachydrine, L-homostachydrine, carotene, phenolic compounds, vanillic acid and p-hydroxybenzoic acid, heterosides, p-coumaric, naringenin, tannic, ferulic, salicylic, sinapic, caffeic and chlorogenic, hesperetin	(72)
*Acacia nilotica*	Flavonoids, phenols, saponins, steroids, tannins	(73)
*Allium cepa* L.	Allyl propyldisulfide as well as S-methyl cysteine sulfoxide	(74)
*Allium sativum* L.	S-allyl cysteine sulfoxide	(75)
*Aloe vera* (L.) Burm. f.	Pseudoprototinosaponin and prototinosaponins	(76)
*Aegle marmelos*	Aegeline	(77)
*Bacopa monnieri* (L.) Wettst.	Herpestine, brahmine, Bacosine and flavonoids	(78)
*Bauhinia variegata* L.	Roseoside	(79)
*Berberis aristata* DC.	Alkaloid, tannins, phytosterols, flavonoid, volatile oils	(80)
*Berberis aristata*	Berberine phenoxide, ketoberberine benzoate A, ketoberberine benzoate B Root—oxyberberine, columbamine, oxyacanthine, berbamine, palmitine, Berberine, jatrorrhizine, tetrahydropalmatine,	(81)
*Catharanthus roseus*	Catharanthine, vindoline, vindolinine	(82)
*Cinnamomum cassia* (L.) J. Presl	Cinnamic acid and cinnamaldehyde	(83)
*Curcuma longa* L.	Curcumin	(84)
*Curcuma longa*	Curcuminoids, bisdemethoxycurcumin	(43)
*Curcuma longa*	Turmerin	(85)
*Erigeron annuus*	Caffeic acid, flavonoids, tannins triterpenoids, saponins, sterols, alkaloids	(86)
*Erigeron annuus*	Erigeroflavanone	(87)
*Erigeron annuus*	3,5-Di-O-caffeoylquinicacid	(88)
*Fagopyrum esculentum*	Rutin and quercetin	(61)
*Ficus bengalensis*	Leucocyandin 3-O-beta-d-galactosyl cellobioside as well asleucopelargonidin-3- O-alpha-L rhamnoside	(77)
*Ficus racemose*	Kaempferol, naringenin, baicalein, quercetin	(43)
*Galega officinalis*	Biguanides	(89)
*Galega officinalis* L.	Querccetin and gaultherin	(58)
*Gymnema sylvestre* (Retz.) R.Br. ex Sm.	Dihydroxy gymnemic triacetate, Gymnemosides A, B, gymnemic acid V, gymnemic acids I–IV and gymnemasaponin	(90)
*Jasminum officinale*	Ascorbic acid alkaloids, resin, carbohydrates, coumarins, flavonoids salicylic acid, saponins, tannins, and terpenoids	(65)
*Mangifera indica*	Mangiferin	(91)
*Mangifera indica*	C-glucosylxanthone mangiferin	(92)
*Momordica angolensis* R. Fern.	Momordicin, charantin, and galactose-binding lectin	(93)
*Morus alba*	Moracin M, steppogenin-4′-Ob-D-glucosiade and mullberroside	(94)
*Morus alba*	Moracin M, steppogenin-4′-O-β-D-glucosiade and mullberroside A	(95)
*Nelumbo nucifera* Gaertn.	Nuciferine	(96)
*Ocimum sanctum* L.	Ursolic acid, ursolic acid stearoyl glucoside	(97)
*Oroxylum indicum* (L.) Kurz	Baicalein	(98)
*Piper longum*	Piperine	(99)
*Piper sarmentosum* Roxb.	Quercetin, naringenin, hesperetin and taxifolin/dihydroquercetin	(84)
*Polygala senega* L.	Z-senegins II and III, IV, esenegasaponin C and Z-senegasaponin C	(100)
*Psidium guajava* L.	Flavonoids, triterpenoids, tannins, saponins, sterols, alkaloids	(101)
*Psidium guajava*	Strictinin, isostrictinin and pedunculagin	(102)
*Psidium guajava*	Morin	(103)
*Psidium guajava*	Strictinin and isostrictinin and pedunculagin	(104)
*Pterocarpus marsupium* Roxb.	Epicatechin	(105)
*Punica granatum*	Punicalagin, valoneic acid dilactone, anthocyanin, phenolic and non-phenolic acids, Glutenins and tannins	(106)
*Punica granatum*	Tannin (cid)	(107)
*Solanum torvum* Sw.	Rutin, caffeic acid, gallic acid and catechi	(108)
*Stephania tetrandra* S. Moore	Tetrandrine 2′- N-β-oxide tetrandrine 2′-N-α-oxide, tetrandrine 2- N- β-oxide, fangchinoline 2′-N-α- oxide, 2′-N-norfangchinoline, and 2′-N-methyltetrandrinium chloride	(109)
*Stevia achalensis* Hieron.	Stevioside, steviol	(110)
*Swertia chirayita*	Swerchiri	(111)
*Syzygium cumini*	Mycaminose	(112)
*Tectona grandis* L.f.	3,8-dihydroxy-2-methyl anthraquinone	(13)
*Teucrium polium* L.	Apigenin	(113)
*Tinospora cordifolia* (Willd.) Miers	Tinosporaside	(114)
*Trigonelia foenumgraecum*	Trigonelline and nicotinic acid	(50)
*Trigonella foenumgraecum*	4-hydroxyleucine and hydroxyisoleucine	(115)
*Triticum aestivum* L.	Polysaccharide	(116)

Documentation of traditional medicinal plants, along with a screening of their biological properties, is a key part of the search for novel drugs to treat drug-resistant pathogens or disorders related to oxidative stress, such as diabetes, in the modern era (40). Certain phytochemicals biological effects, such as antidiabetic, anticancer, antiviral, antimicrobial, antibacterial, and antioxidant activity, have been found to be valuable in the cure of diabetes and other ailments. Numerous phytochemicals and plant extracts confirmed the effectiveness of diabetes (41). The existence of pathogenic organisms or disease-causing processes, such as cancer, diabetes, and inflammatory illnesses, is intimately associated with free radical oxidative (42).

## 6 Ethnopharmacological research of specific plant species used for the control of diabetes

As mentioned in Table 2, the therapeutic benefits of plants are related to the existence of many bioactive compounds that are a good start for diabetes (43). The traditional uses of plants and the effect of different plant extract components show antidiabetic activities (44), while, some plants assist in the discovery of compounds that aid in the production of synthetic drugs to treat diabetes (45). For the practical use of these plants to treat diabetes, ethnopharmacological studies are required (46). Various *in-vivo* trials have been showing a demand to assess the ethnomedicinal significance of the plant’s role in treating diabetes (47). So, Table 3 provides information about various plant species, biochemicals, the dose taken, the experimental model (animal model), compounds as they control hyperglycemia, and their efficiency against diabetes.

**Table 3:** In-vivo data of most common ethnomedicinal plants in Himachal Pradesh used for the treatment of diabetes.

Plant species	Major phytochemicals	Model used	Induction of diabetes	Dose	References
*Asparagus racemosus*	Alkaloid, flavonoid and phenols	Wistar rats	Streptozotocin (STZ)	200, 400	(117)
*Acacia arabica*	Polyphenols, tannins, and flavonoids	Rat	STZ	100, 200	(118)
*Acacia nilotica* (L.)	Alkaloids	Rats	STZ	100	(119)
*Acacia nilotica* (L.) Delile	Flavonoid, phenols, alkaloids	Rats	Alloxan	50	(120)
*Achyranthes aspera*	Alkaloid. flavonoids, phenols	Rats	STZ	1,000	(121)
*Aegle marmelose*	Alkaloid, flavonoids, phenols	Rats	STZ	125, 250	(122)
*Afzelia africana*	Alkaloids, flavonoids, phenols, tannins, saponins, terpenoids	Rats	STZ	100, 200	(123)
*Allium cepa*	Flavonoids, phenols	Rats	STZ-	100	(124)
*Allium cepa* L.	Phenolic compounds polysaccharides, saponins	Rats	STZ	500	(125)
*Allium sativum*	Flavonoids, phenols	Rats	STZ	100	(126)
*Allium sativum* L.	Flavonoids, alkaloids, polyphenols,	Rats and mice	STZ and alloxan	100	(127)
*Aloe barbadensis* Miller	Polyphenols, tannins, flavonoids	Rats	STZ	300, 500	(128)
*Aloe vera* L.	Polyphenols, tannins, flavonoids	Rats	STZ	100, 200	(129)
*Amaranthus caudatus*	Flavonoids phenols	Rats	STZ	200, 400	(130)
*Amaranthus esculantus*	Tannins, alkaloids, flavonoids, phenols, saponins terpenoids	Rats	STZ	100, 200	(131)
*Andrographis paniculata*	Alkaloids, flavonoids, phenols, tannins, saponins terpenoids	Rats	Alloxan	100, 200	(132)
*Azadirachta indica* A. Juss	Flavonoid, tannins, phenols, alkaloids	Albino wistar rats	Alloxan	200, 400, 800	(133)
*Bauhinia variegata*	Flavonoids phenols	Rats	Alloxan and STZ	300, 400, 500	(80)
*Berberis aristata* DC	Flavonoid, tannins, phenols, alkaloids	Male albino Wistar rats	Alloxan	200	(57)
*Berberis vulgaris* L	Tannins, alkaloids, saponins, phytosteroidsanthraquinones	Male Wistar rats	STZ	250	(134)
*Cajanus cajan* L.	Phenols	Swiss albino mice	Alloxan	200, 400	(135)
*Cassia auriculata*	Alkaloids, flavonoids, phenols, tannins, saponins terpenoids	Rats	STZ	400	(5)
*Cassia fistula* Linn	Alkaloids, glycosides, flavones, tannins, terpenes, sterols, saponins	Rats	STZ	200, 400	(134)
*Curcuma aromatica*	Phenols, flavonoids	Wister albino rats	STZ	200	(136)
*Curcuma longa*	Polyphenols, tannins, flavonoids	Albino rats	Alloxan	400	(137)
*Curcuma longa*	Alkaloids, glycosides, saponins, steroids, flavonoids	Mice	Alloxan	100, 200	(138)
*Cymbopogon citratus* (DC.) Stapf	Alkaloids, flavonoids, phenols	Rats	STZ	500	(119)
*Ficus bengalensis*	Alkaloids, terpenoids, quinones, flavonoids	Rats	Alloxan	100	(94)
*Gymnema sylvestre*	Alkaloids, phenols, tannins, saponin	Rats	Alloxan	100, 200, 300	(139)
*Juglans regia*	Alkaloids, flavonoids, phenols, tannins, saponins, terpenoids	Rat	STZ	250, 500	(138)
*Mangifera indica*	Flavonoid, phenols	Rats	STZ	100	(138)
*Nelumbo nucifera*	Flavonoids, phenols	Rats	STZ	100	(140)
*Ocimum basilicum* L.	Flavonoids, alkaloids, polyphenols,	Rats	STZ	200, 250	(141)
*Ocimum sanctum*	Polyphenols, tannins, flavonoids	Male albino Wistar rats	Alloxan	200	(142)
*Ocimum sanctum* L.	Saponin, flavonoids, triterpenoids, tannins	Rats	STZ	100, 200	(143)
*Picrorhiza kurroa* Royl	Cucurbitacin, phenols, flavonoids	Male Wistar rats	STZ	100, 200	(144)
*Polygala senega*	Flavonoids, alkaloids, polyphenols,	Mice	STZ	50, 100	(145)
*Swertia chirayita*	Flavonoids, terpenoids	Rats	STZ	(100, 200	(30)
*Swietenia macrophylla*	Tannins alkaloids, flavonoids, phenols, saponins, terpenoids	Rats	STZ	200, 300	(146)
*Syzygium cumin*	Flavonoids, phenols	Mice and Rats	STZ	100, 200	(147)
*Syzygium cumini*	Terpenoids, alkaloids, flavonoids, phenols, tannins, saponins	Rats	STZ	100	(148)
*Tectona grandis* L.	Alkaloids, glycosides, saponins, steroids, flavonoids	Rats and mice	Alloxan	100	(149)
*Tinospora cordifolia*	Flavonoids, phenols	Rats	Alloxan	100 and 200	(150)
*Tinospora cordifolia*	Alkaloids, terpenoids, quinones, flavonoids	Rats	STZ	250	(151)
*Tinospora cordifolia*	Alkaloids, phenols, tannins, saponin	Rats	STZ	200, 400	(150)
*Withania somnifera*	Flavonoids, alkaloids, polyphenols,	Rats	STZ	100, 200	(152)
*Withania somnifera* L.	Flavonoids	Male albino Wistar rats	Alloxan	100, 200	(153)
*Zingiber officinale*	Tannins, alkaloids, flavonoids, phenols, saponins, terpenoids		STZ	100	(154)

## 7 Conclusion

Long-term use of allopathic drugs has been associated with serious side effects and metabolic complications. The present review highlights the ethnomedicinal diversity of Himachal Pradesh, identifying key antidiabetic plants such as *Syzygium cumini*, *Momordica charantia*, *Tinospora cordifolia*, *Gymnema sylvestre*, *Aegle marmelos*, and *Trigonella foenum-graecum*. Their efficacy is attributed to bioactive compounds including mycaminose, charantin, momordicin, tinosporaside, gymnemic acids, aegeline, and 4-hydroxyisoleucine, which regulate glucose metabolism through insulin secretion, enhanced glucose uptake, and enzyme inhibition. The findings demonstrate that these bioactive compounds regulate glucose metabolism through multiple mechanisms, including enhancement of insulin secretion and reduction of intestinal glucose absorption. The *in-vivo* studies presented in this review show that many plants, including *Asparagus racemosus*, *Acacia nilotica*, *Aegle marmelos*, *Allium sativum*, *Gymnema sylvestre*, and *Tinospora cordifolia*, have high antidiabetic potential when tested in models of diabetes induced by streptozotocin and alloxan with dosing concentrations of around 100–400 mg/kg, mostly due to bioactive compounds such as flavonoids, alkaloids, phenols, and saponins. Unlike previous descriptive studies, this review integrates ethnomedicinal knowledge with phytochemical and pharmacological evidence, providing a structured foundation for herbal drug discovery. It is essential as this type of information links conventional ethnomedicinal data with current phytochemical and pharmacological information, providing a systematic framework for herbal medicine research. This paves the way for the discovery of potential bioactive substances and guides researchers toward conducting further research in areas where there are gaps in information. Furthermore, this study identifies key research gaps, including the need for molecular docking studies, target validation, and advanced *in-vivo* investigations to support clinical translation.
